# Outcomes of Babies with Opioid Exposure (OBOE): protocol of a prospective longitudinal cohort study

**DOI:** 10.1038/s41390-022-02279-2

**Published:** 2022-08-30

**Authors:** Carla M. Bann, Jamie E. Newman, Brenda Poindexter, Katherine Okoniewski, Sara DeMauro, Scott A. Lorch, Deanne Wilson-Costello, Namasivayam Ambalavanan, Myriam Peralta-Carcelen, Catherine Limperopoulos, Kushal Kapse, Jonathan M. Davis, Michele Walsh, Stephanie Merhar

**Affiliations:** 1 RTI International, Research Triangle Park, NC USA; 2 Emory University, Atlanta, GA USA; 3 Children’s Hospital of Philadelphia, Philadelphia, PA USA; 4 Case Western Reserve University, Cleveland, OH USA; 5 University of Alabama, Birmingham, AL USA; 6 Children’s National Medical Center, Washington, DC USA; 7 Tufts University, Boston, MA USA; 8 Eunice Kennedy Shriver National Institute of Child Health and Human Development, Bethesda, MD USA; 9 Cincinnati Children’s Hospital Medical Center and University of Cincinnati Department of Pediatrics, Cincinnati, OH USA

## Abstract

**Background:**

While the health, social, and economic impacts of opioid addiction on adults and their communities are well known, the impact of maternal opioid use on the fetus exposed in utero is less well understood.

**Methods:**

This paper presents the protocol of the ACT NOW Outcomes of Babies with Opioid Exposure (OBOE) Study, a multi-site prospective longitudinal cohort study of infants with antenatal opioid exposure and unexposed controls. Study objectives are to determine the impact of antenatal opioid exposure on brain development and neurodevelopmental outcomes over the first 2 years of life and explore whether family, home, and community factors modify developmental trajectories during this critical time period.

**Results:**

Primary outcomes related to brain development include cortical volumes, deep cerebral gray matter volumes, resting-state functional connectivity measures, and structural connectivity measures using diffusion tensor imaging. Primary neurodevelopmental outcomes include visual abnormalities, cognitive, language, and motor skills measured using the Bayley Scales of Infant Development and social–emotional and behavioral problems and competence measured by the Brief Infant-Toddler Social and Emotional Assessment.

**Conclusions:**

The OBOE study has been designed to overcome challenges of previous studies and will help further understanding of the effects of antenatal opioid exposure on early infant development.

**Impact:**

- This study will integrate MRI findings and comprehensive neurodevelopmental assessments to provide early insights into the functional topography of the brain in this high-risk population and assess MRI as a potential biomarker.
- Rather than conducting neuroimaging at a single time point, the study will include serial MRI assessments from birth to 2 years, allowing for the examination of trajectories throughout this period of rapid brain development.
- While previous studies often have had limited information on exposures, this study will use umbilical cord assays to accurately measure amounts of opioids and other substances from 20 weeks of gestation to birth.

## Introduction

Neonatal opioid withdrawal syndrome (NOWS) has emerged as a generational byproduct of the opioid epidemic. Following the increase in opioid use by pregnant mothers,^1,2^ the incidence of NOWS has also continued to increase with recent prevalence estimates observing growth from 4.0/1000 births in 2010 to 7.3/1000 births in 2017.^3^

The effects of maternal opioid use on the fetal brain are largely unknown. Basic science studies indicate multiple negative effects of in utero opioid exposure on brain development, including decreased neurotransmitter levels, decreased neurogenesis, increased apoptosis, and altered myelination.^4–6^ Human imaging studies suggest that children exposed to opioids in utero have smaller regional brain volumes^7–9^ and altered structural connectivity as compared with unexposed children.^7,8,10,11^ Functional connectivity also appears to be altered in infants with prenatal exposure to opioids and other drugs.^12–14^ However, previous magnetic resonance imaging (MRI) studies on the brain development of opioid-exposed infants and children have been small and may not have used appropriate control groups. It is therefore unknown whether these potential effects seen in the brains of opioid-exposed children are related to opioids themselves or to other confounding factors such as polysubstance use, nicotine exposure, maternal mental health disorders, or environmental factors, such as poverty.

Studies on the developmental and behavioral outcomes of opioid-exposed infants and children have shown inconsistent results. Although there are trends toward lower performance on standardized assessments such as the Bayley in exposed children at 2 years of age, the differences may not be clinically significant.^15–17^ Although several studies have shown that opioid-exposed children may have a higher risk of reduced visual function, strabismus, and nystagmus,^18–20^ no large-scale studies have evaluated visual outcomes in this population. Behaviorally, children exposed to buprenorphine have shown difficulties with working memory, hyperactivity, impulsivity, and attention with increased risk for attention-deficit/hyperactivity disorder overall.^21,22^ Additional risks present later in life as well with emerging conduct and adjustment disorder, along with a greater likelihood of diagnosis of anxiety, emotional disturbances, and autism spectrum disorder.^23^ However, a well-established set of literature has demonstrated that after controlling for social–environmental and socioeconomic variables, the impact of in utero exposure is no longer significant for many developmental and behavioral outcomes.

Previous studies have significant limitations that must be addressed with rigorous study designs. Although there are indications that NOWS can have significant short- and long-term effects, high-quality research to document the profile of infants with NOWS through childhood and into adulthood is lacking. A particular challenge is the heterogeneity within the population of infants with NOWS.^24^ A variety of factors such as multiple in utero exposures (e.g., additional illicit drugs, alcohol, and tobacco), duration and amount of exposure, medication-assisted treatment (e.g., buprenorphine, methadone), and severity of neonatal withdrawal may lead to variance in developmental profiles across the population and limited accuracy, validity, and generalizability of findings.^24^

This article outlines the protocol for the Advancing Clinical Trials in Neonatal Opioid Withdrawal Syndrome (ACT NOW) Outcomes of Babies with Opioid Exposure (OBOE) Study, a prospective longitudinal cohort study of the outcomes of infants with antenatal opioid exposure and controls from birth to 2 years of age (Clinical Trials.gov NCT04149509). The OBOE study will address limitations of prior research in the following ways:

- Collecting accurate and comprehensive data about infant exposure to opioids and other substances using both maternal report and umbilical cord toxicology at the time of birth in both exposed infants and controls.
- Prospectively recruiting control infants from the same birth hospitals as opioid-exposed infants.
- Obtaining a measure of maternal IQ and information about maternal mental health disorders to adjust for effects of maternal IQ and maternal anxiety/depression on child development.
- Collecting neuroimaging data in the first month of life to minimize effects of the postnatal environment on MRI findings.
- Collecting information about the trajectory of brain, development, and behavior with serial neuroimaging and developmental and behavioral assessments at several time points.
- Collecting information about parenting and the child’s environment via a home visit to assess the impact of the home environment on brain, behavior, and development.

## Methods

### Study aims and hypotheses

The OBOE Study will address three specific aims. The first aim is to determine the impact of antenatal opioid exposure on brain structure and connectivity over the first 2 years of life. We hypothesize that antenatal opioid exposure reduces cortical and deep cerebral gray matter volumes and disrupts the structural and functional connectome as measured by diffusion tensor imaging (DTI) and resting-state functional connectivity MRI over the first 2 years of life. The second aim is to define medical, developmental, and behavioral outcomes over the first 2 years of life in infants exposed to opioids. Our hypothesis is that the magnitude of antenatal opioid exposure and co-exposures with other psychotropic drugs will independently affect developmental and behavioral outcomes. The third aim is to explore whether and how the home environment, maternal mental health, and parenting modify trajectories of brain connectivity and neurodevelopment over the first 2 years of life. For this aim, we hypothesize that several identifiable and modifiable postnatal factors mediate the effect of antenatal opioid exposure and jointly contribute to poorer neurodevelopmental outcomes.

### Study sites

**Fig. 1:**
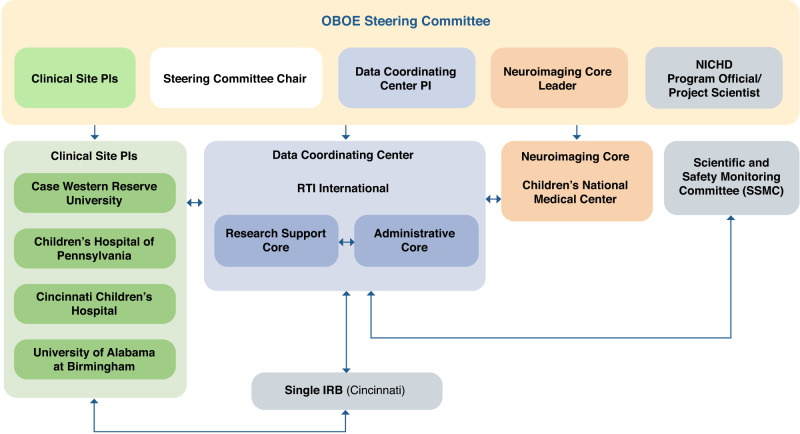
Organization of the ACT NOW OBOE Study. Organization of the ACT NOW OBOE Study including Steering Committee, Clinical Sites, Data Coordinating Center, Neuroimaging Core, Scientific and Safety Monitoring Committee, and single IRB.

The organization of the ACT NOW OBOE Study is shown in Fig. 1. The study includes four clinical sites [Case Western University, Children’s Hospital of Pennsylvania (CHOP), Cincinnati Children’s Hospital Medical Center (CCHMC), and University of Alabama at Birmingham], a data coordinating center (DCC) at RTI International, and a Neuroimaging Core at Children’s National Medical Center. The clinical sites were chosen based on an extensive history of coordinated consortium work through the well-established *Eunice Kennedy Shriver* National Institute of Child Health and Human Development Neonatal Research Network with high rates of recruitment enrollment, protocol compliance, and follow-up.^25^ Through a single Institutional Review Board at CCHMC, all contributing clinical sites, the Neuroimaging Core, and the DCC at RTI received approval for human subjects’ research activities for this study and informed consent is obtained for all participants.

### Study population

A total of 300 infants will be enrolled in the study. The OBOE study will include infants with antenatal opioid exposure and unexposed controls, resulting in 200 opioid-exposed and 100 unexposed infants. Infants will be enrolled at the clinical sites, including a wide range of racial, ethnic, and socioeconomic populations. All birthing mothers at each participating clinical site hospital will be screened for study eligibility.

#### Inclusion criteria

Opioid-exposed infants will be included if born at or after 37 weeks gestation (to eliminate the impact of prematurity on neurodevelopmental outcomes) with second or third trimester opioid exposure as determined by maternal history; maternal urine toxicology screen at delivery; or infant urine, meconium, or umbilical cord toxicology screen. Control infants will be included if born at or after 37 weeks gestation with no antenatal drug exposure as determined by maternal history and/or maternal urine toxicology screen at delivery. Control infants will be matched to exposed infants based on clinical site and date of birth up to 120 days after the date of birth of the exposed infant. Understanding that demographics and exposures may vary in the opioid-exposed and control groups, we will assess demographics once we reach 33% enrollment and consider matching for factors such as maternal education and use of tobacco or other substances.

#### Exclusion criteria

Infants for both groups will be excluded from participation based on the following criteria: (1) infants with known chromosomal or congenital anomalies potentially affecting the central nervous system; (2) Apgar score at 5 min of <5; (3) any requirement for positive pressure ventilation in the Neonatal Intensive Care Unit (NICU); (4) inability to return for outpatient MRI or follow-up, (5) intrauterine growth restriction below the third percentile, and (6) heavy alcohol use during pregnancy (eight or more alcoholic drinks per week).

### Study procedures

#### Screening and enrollment

Infants will be prescreened by the research coordinator in birth hospitals using the electronic medical record systems. Parents/guardians of eligible infants will be approached for participation and the research coordinator will obtain written informed consent. Participant incentives are outlined in the consent form. If the infant is in the custody of the county at discharge, the research coordinator will discuss the study and obtain consent from the case worker or supervisor. The research coordinator will also verbally discuss with the foster family who has physical custody of the infant to ensure that they are willing to facilitate the infant’s participation.

#### Measures and data collection

Following eligibility confirmation and consent, data collection will begin through a series of direct assessment, parent/caregiver report, and neuroimaging from 0 to 1 month through 24 months of age at 6-month intervals both in person (e.g., in hospital/clinic setting, at home) and virtually (e.g., telephone interviews). A comprehensive battery of assessments and the timeline for administration are provided in Table 1.

**Table 1:** Battery of assessments and timing of administration.

Assessment	Months				
	0–1	6	12	18	24
Child outcomes					
Direct child administration					
Magnetic Resonance Imaging (MRI)	●	●			●
NICU Network Neurobehavioral Scale, Second edition (NNNS-II)	●				
Developmental Assessment of Young Children, Second Edition (DAYC-2)			●		
Bayley Scales of Infant and Toddler Development, Fourth Edition (Bayley-4)					●
Physical/neurological exam including gross motor function classification					●
Spot^TM^ Vision Screener					●
Parent/caregiver report					
Infant medical history	●	●	●	●	●
Brief Infant Sleep Questionnaire (BISQ)			●		
Brief Infant-Toddler Social and Emotional Assessment (BITSEA)			●		●
Modified Checklist for Autism in Toddlers, Revised with Follow-Up (M-CHAT-R/F)					●
Effect modifiers					
Maternal/family					
Maternal medical history	●				
Maternal substance use	●				
OBOE stigma scale	●				
Adverse Childhood Experience questionnaire (ACE)	●				
Maternal Postnatal Attachment Questionnaire (MPAQ)		●			
Parent-Reported Outcome Measure Information System (PROMIS) Measures	●	●		●	●
Parenting Stress Index, Fourth Edition-Short Form (PSI-4-SF)		●		●	●
Wechsler Abbreviated Scale of Intelligence, Second Edition (WASI-II)	●				
Home/community					
Socioeconomic status (including food insecurity and housing instability)	●	●	●	●	●
Home Observation Measurement of the Environment (HOME)			●		
Parent/caregiver report of neighborhood safety and support	●	●	●	●	●
Zip code-based indicators of social determinants of health	●	●	●	●	●

Parental/caregiver response questionnaires are estimated to take approximately 60 min and are inclusive of anywhere from 80 to 180 questions based on time point of administration. Investigators were consistently attentive to the burden presented to participants and strategically selected measures and administration time points to include only key questions during protocol development. Procedures inclusive of opportunities to complete forms via different modalities (e.g., virtual/online completion, phone interview, in-person), while their child is participating in developmental assessment or scanning, along with careful training of examiners (e.g., encouraging proper wait time for reflecting, explaining participants can skip particularly triggering or difficult questions) will be utilized to help decrease perceptions of burden or stress when interacting with these assessment measures.

##### Antenatal drug exposure

To identify potential antenatal drug exposures, clinical site personnel will collect a segment of each infant’s umbilical cord. The cords will be analyzed by the CCHMC Mass Spectrometry Laboratory, using a panel testing for 45 drugs of abuse and cotinine (a nicotine metabolite). In addition, biological mothers are asked a series of questions on medication and substance use during pregnancy, including the type of substance and the frequency, amount, and trimester when used. We will document whether mothers participated in an opioid treatment program at any point during pregnancy and type (if any) of medication prescribed.

##### Sociodemographic characteristics

At each time point, caregivers will be asked to provide information regarding socioeconomic status (SES), including caregiver education and employment, child’s medical insurance, living arrangements, household income, housing stability, food insecurity, and neighborhood safety. Items included in the SES form were based on common data elements from prior research studies and national surveys, such as the American Community Survey. Food insecurity is assessed using the two-item Hunger Vital Sign™ screener^26^ recommended by the American Academy of Pediatrics and the Food Research and Action Center.^27^ In addition, at the 0- to 1-month visit, information on maternal medical history will be obtained, including pregnancy complications, medical conditions, and psychiatric disorders and medications. We will also document whether the mother had adequate prenatal care, defined as having three or more visits and prenatal care starting prior to the third trimester.

##### Growth and medical conditions

A detailed neonatal chart review will also be conducted for all infants, including sex, gestational age, birth weight, birth length, birth head circumference, race, ethnicity, Apgar scores, and length of hospital stay. In addition, for opioid-exposed infants, data will be collected on Neonatal Abstinence Scoring System used, Finnegan scores (measure of neonatal withdrawal), neonatal toxicology screen results, medications used for the treatment of NOWS (including dose and duration of treatment), and custody at discharge.

Interval medical history including rehospitalizations, infant feeding and growth, immunizations, new diagnoses, and services will be assessed at 6, 12, 18, and 24 months. Additionally, instrument-based vision screening will be performed at 24 months with the Welch Allyn Spot Vision Screener, a tool that detects risk factors associated with amblyopia, such as myopia, hyperopia, astigmatism, anisometropia, gaze, and anisocoria.

##### Neuroimaging

Infants will undergo an MRI scan at three time points during study participation (0–1, 6-, and 24-month time points), with each site using a 3 T MRI scanner. The “feed and swaddle” technique will be used to optimize natural sleep and eliminate the need for sedation. This technique includes infants being fed immediately before the exam, undressed to a diaper, swaddled in a blanket and MedVac bag (CFI Medical Solutions, Fenton, MI), and positioned in the scanner. Ear plugs and Minimuffs (Natus Medical Incorporated, San Carlos, CA) will be applied for hearing protection. For the 6- and 24-month scans, previously published^28^ methods will be used, including (1) scheduling scans in the evening hours around the infant’s bedtime or during naptime for the 6-month-olds; (2) skipping daytime naps the day of the scheduled imaging session; (3) allowing time for the family to get the infant to sleep in a private testing room outside the MRI suite and then waiting for 15–20 min before moving the infant to the scanner; (4) reducing acoustic noise levels by using earplugs, Minimuffs, headphones, and sound-insulating foam inserts in the scanner; (5) using a larger MedVac immobilization bag for the toddlers and securing after the child falls asleep; and (6) designing the MRI protocol to require <30 min to ensure uninterrupted sleep during the scan. Research staff will be in visual contact with infants throughout the MRI exam. Total MRI scan time for each time point should be approximately 60 min. MRI protocols are provided in Table 2.

**Table 2:** MRI specifications by sequence and type of scanner.

Component	Siemens (Prisma)				Philips (Ingenia)			
	T1	T2	Diffusion	fMRI	T1	T2	Diffusion	fMRI
TR (ms)	1600	3200	3440	1000	9.5	2500	2329	1000
TE (ms)	3.01	492	98.4	30	4.5	252	102	30
TI (ms)	1000	N/A	N/A	N/A	1000	N/A	N/A	N/A
Resolution (mm)	1 × 1 × 1	1 × 1 × 1	2.5 × 2.5 × 2.5	3 × 3 × 3	1 × 1 × 1	1 × 1 × 1	2.5 × 2.5 × 2.5	3 × 3 × 3
Flip angle (deg)	15	T2 Var	78	80	8	90	90	80
Parallel imaging	2×	2×	Off	Off	2×	2×	Off	Off
MultiBand acceleration	Off	Off	3	4	Off	Off	3	4
Diffusion directions	N/A	N/A	48	N/A	N/A	N/A	48	N/A
*b*-values	N/A	N/A	1000	N/A	N/A	N/A	1000	N/A
Acquisition time	3:26	2:45	3:10	7:01	3:20	3:05	2.10	7:01

The MRI protocol will be harmonized across all four clinical sites. Structural scans will be read by a pediatric neuroradiologist, and abnormal findings will be reported to the infant’s family and pediatrician as dictated by each institution’s policy.

##### Neurodevelopmental assessments

The following neurodevelopmental assessments will be administered directly to the infant/toddler and supplemented with parent report and child observation at the 12- and 24-month visits to measure attainment of developmental skills across a variety of domains. The NICU Network Neurobehavioral Scale, Second Edition (NNNS-II)^29^ will be administered at the 0- to 1-month visit, to provide insight into neurobehavior in the neonatal period. At 12 months, the *Developmental Assessment of Young Children, Second Edition* (DAYC-2)^30^ cognition, communication, and physical development protocols will be administered via parent interview and observation. At 24 months, the *Bayley Scales of Infant and Toddler Development, Fourth Edition*^31^ and a standardized neurologic exam will be completed. If an infant has an abnormal neurologic exam, the Gross Motor Function Classification System classification will be used to describe the level of ability.^32^ Results would then be shared with the infant’s family and medical team and if appropriate, referrals to applicable support programs provided.

##### Behavioral assessments

Considering antenatal exposure to substances and the potential impact on a child’s social–emotional and behavioral skills and future mental health, three parent report measures will be used at the 12- and 24-month visits. At 12 months, the infant’s sleep will be assessed by the Brief Infant Sleep Questionnaire (BISQ).^33^ The *Brief Infant-Toddler Social and Emotional Assessment* (BITSEA)^34,35^ will be administered at the 12- and 24-month visits to examine potential social-emotional/behavioral difficulties. Additionally, the Modified Checklist for Autism in Toddlers-Revised with Follow-Up (M-CHAT-R/F)^36^ will be administered at 24 months to identify signs associated with autism spectrum disorder and/or developmental delay.

##### Psychosocial assessments

Maternal mental health including stress and depression, along with maternal cognitive functioning, experience of social risk factors, parenting style, parent–child interaction patterns, SES, and caregiver education can all have a potentially substantial impact on early childhood development. These specific areas will be targeted for further investigation within the OBOE Study. To capture SES, a questionnaire was developed for this study that includes information including income, caregiver education, employment status, household composition, housing instability, food insecurity, and participation in the foster care system.

The Parent-Reported Outcome Measure Information System (PROMIS)^37^ will be used to better understand parental mental health needs. PROMIS is a set of measurements that examines a wide range of functioning in children and adults. The OBOE study will focus on anxiety (PROMIS Short Form v1.0 - Anxiety - 8a 31May2019), depression (PROMIS_SF_v1.0_-_ED-Depression_8a_5-31-2019), anger (PROMIS Short Form v1.1 - Anger - 5a 27Apr2016), life meaning and purpose (PROMIS Short Form v1.0 - Meaning and Purpose - 8a 18Jul2017), and social support (PROMIS v2.0 - Emotional Support Short Form 4a 23June2016). Additionally, perceived levels of parenting stress will be captured by caregiver report at 6, 18, and 24 months through the *Parenting Stress Index, Fourth Edition-Short Form* (PSI-4-SF).^38^

A profile of maternal/primary caregiver background will be captured through direct testing and report. The Wechsler Abbreviated Scale of Intelligence, Second Edition (WASI-II)^39^ Verbal and Matrix Reasoning subtests will be administered to the mother by a trained coordinator during the 0- to 1-month MRI visit to examine cognitive functioning, an established correlate to childhood cognitive and language outcomes. The Adverse Childhood Experiences (ACE)^40^ will be used to measure specific childhood experiences correlating with future social risk factors and negative health outcomes, completed by the mother at the 0- to 1-month MRI visit (or at the 6-month visit if not done at 0–1 months).

Aspects of the child–caregiver relationship and environment will be assessed via caregiver report and observation. Caregivers will complete the Maternal Postnatal Attachment Questionnaire (MPAQ)^41^ to measure the quality of interactions in the domains bonding, absence of hostility, and pleasure in interaction during the 6-month visit. During the in-home 12-month visit, examiners will complete the *Home Observation for Measurement of the Environment* (HOME).^42^ The HOME^42^ is a comprehensive assessment of nurturance and stimulation in the home environment based on observation and caregiver interview. Should a home visit not be feasible or if the parents refuse the home visit, a version of the HOME assessment which has modified for administration virtually through a video visit will be used.

### Data analyses

#### MRI data processing and analysis

##### Image preprocessing and brain segmentation for volumetric analysis

All MRI images will be processed using the following steps: (1) T2-weighted MR image of each subject will be linearly aligned onto their corresponding T1-weighted image; (2) brain extraction will be performed using a learning-based algorithm;^43^ (3) intensity inhomogeneity will be corrected;^44^ (4) T1- and T2-weighted images will be normalized to zero mean and unit variance; and (5) images will be segmented into white matter, cortical gray matter, lateral ventricle, external cerebrospinal fluid, and deep gray matter. Volumetric analysis will be conducted based on the segmented brain structures.^45,46^

##### Structural connectivity/DTI analysis

At each time point (0–1 month, 6 months, 24 months), a single-subject brain with median brain size and relatively straight medial longitudinal fissure will be used as a single-subject template for inter-subject registration and manual labeling. All neural structures will be delineated in axial images with high contrast from DTI and the anatomical information from DTI tractography, followed by adjustment in coronal and sagittal images.^47,48^ Graphic metrics will also be computed. Primary brain connectome outcome measures will include global graph measures, including cost and global efficiency, and local/regional graph measures, including local efficiency and clustering coefficient.

##### Resting-state functional connectivity MRI analysis

Resting-state functional connectivity MRI will be used to noninvasively assess brain function by simultaneously probing multiple brain networks using the contrast induced by differences in blood oxygenation. Resting-state data will be preprocessed using previously validated techniques that minimize the influence of noise (i.e., head motion, physiologic noise, and scanner noise) on measured blood oxygen-level-dependent (BOLD) signals.^49^ To assess functional connectivity, we will use seed-based correlation methods and graph theoretic techniques. A priori cortical, subcortical, and cerebellar regions of interest (ROIs) will be defined using automatic anatomic labels optimized for neonates and very young children.^50^ These ROIs will be defined in T1/T2 anatomical images and registered to Echo-planar imaging data. A study-specific template will be created for the entire cohort and functional images will be normalized to this template. BOLD time series measured from each ROI will be correlated (i.e., Pearson correlation) to signals obtained from other regions. Changes in connectivity strength and connectivity patterns over time will be measured. To complement this approach, we will also use graph theoretic techniques.

#### Statistical analysis plan

For neuroimaging outcomes (Aim 1), linear and generalized linear mixed-effect regression models will be fit using data from the MRI scans at 0–1, 6, and 24 months to compare trajectories of brain development measures (e.g., cortical and deep cerebral gray matter volumes) over time in infants with and without opioid exposure, controlling for study site and child and family characteristics. The test of a time-by-exposure status interaction will allow us to determine if trajectories over time differ between exposed and unexposed infants. The model will be selected based on the type of outcome with linear models for normally distributed continuous outcomes and generalized linear models for categorical or non-normally distributed continuous outcomes. In addition to the comparisons by the dichotomous opioid exposure variable (i.e., exposed versus unexposed), similar models will examine the impact of the level of opioid exposure on neuroimaging outcomes.

For neurodevelopmental and behavioral outcomes (Aim 2), linear mixed-effect models will be used for continuous outcomes (e.g., Bayley scores) and generalized linear mixed-effect models for categorical outcomes (e.g., presence of a diagnosis), accounting for study site and infant and family characteristics. These models allow us to statistically account for clustering of subjects within study sites. In addition, for outcomes where measurement error is likely to occur, such as different domains of behavioral assessments, latent variable approaches (i.e., structural equation modeling) will allow us to combine more than one measure into a single underlying latent variable to reduce measurement error in models. These models permit testing of hypotheses that, compared with unexposed controls, children with opioid exposure are more likely to have low cognitive scores, demonstrate increased motor abnormalities, and have more behavioral problems, controlling for other possible contributing factors (e.g., other antenatal exposures, postnatal environment, demographics). Sensitivity analyses will be conducted to examine the impact of including subjects with other antenatal exposures in the models.

**Fig. 2:**
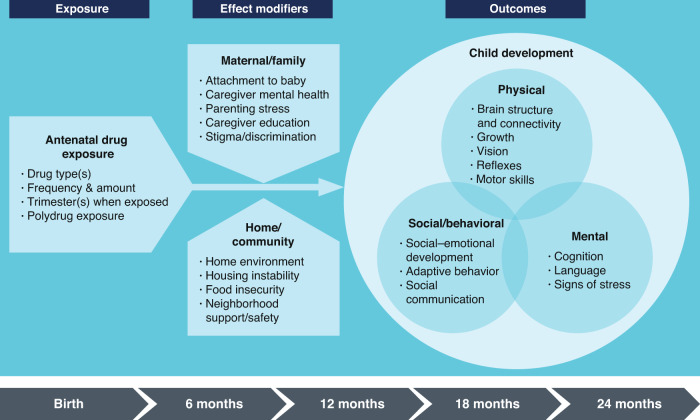
Conceptual Framework of the ACT NOW OBOE Study. Conceptual framework of the relationships between exposures, effect modifiers, and outcomes.

For Aim 3, possible moderators (e.g., family and home factors) of antenatal opioid exposure effects on outcomes will be evaluated as shown in Fig. 2. Using the mixed-effect and SEM models described above, we will test for interactions between opioid exposure and potential moderators/confounders to determine whether opioid exposure has a differential impact on infant outcomes for some subgroups compared to others. For example, we can test whether antenatal opioid exposure effect is amplified among infants who also have a poor home environment.

Missing data are possible for any longitudinal study and are a special cause of concern for the population to be enrolled in this study. We will closely monitor attrition and conduct logistic regression models to compare the characteristics (e.g., opioid exposure, site, maternal and family characteristics) of subjects who remain in the study at 2 years versus those who dropped out to determine if data are missing at random. Patterns of item-level missing data will be examined to determine if particular types of questions (e.g., questions related to maternal drug use) have greater levels of missing data or have missing data rates that differ across groups, suggesting that they may not be reliable. The impact of missing data of both types will be minimized through use of appropriate likelihood-based estimators.^51–53^ These methods yield unbiased estimates and accurate standard errors without sacrificing cases (and thus maximizing statistical power) when data are missing completely at random or predicted by other variables in a given model but independent of the potential values of the outcome itself (i.e., missing at random). If subjects do not appear to be missing at random, we will use pattern mixture models to account for the informative attrition.^54^

#### Sample size and power estimates

Given the paucity of definitive studies on effects of antenatal opioid exposure to 2 years, power calculations reflect a scientifically meaningful effect size of half a standard deviation unit for a longitudinal outcome based on a sample size of 300 (200 exposed infants and 100 unexposed infants). We assumed three repeated measures (0–1, 6, 24 months), with 80% follow-up at 24 months, a conservative intra-cluster correlation of 0.5 for repeated measures, and a two-sided test with a conservative Type 1 error of 0.01 in recognition of the multiple aims specified for our analysis.

With a sample size of 300, the study will have statistical power of 99% to detect effects this size (*d* = 0.5) with statistical significance. A smaller effect size of a third of a standard deviation (*d* = 0.33) is still detectable with 98% power. Power will be lower but respectable to detect meaningful differences in binary outcomes. Using the proportion of head circumference below the 10th percentile as an example, under the same assumptions, shows that for a control group proportion of 10%, there is 82% power to detect proportions in excess of 25% in the opioid-exposed group (relative risk of 2.5 or higher).

The cross-sectional analyses have lower but still respectable power, especially for continuous outcomes such as the 24-month Bayley cognitive score. As before, for a scientifically meaningful effect size of half a standard deviation unit, based on the sample size of 300 with 80% follow-up at 24 months, a conservative two-sided test at Type I error of 0.01 has 85% power to detect effects of this size with statistical significance. Not surprisingly, only large effects of antenatal opioid exposure (relative risk of 3.7 or higher) are detectable for binary outcomes with low (5%) prevalence in the control/unexposed infants.

## Discussion

The ACT NOW OBOE Study is a longitudinal cohort study designed to prospectively examine the neurodevelopmental, behavioral, and social/family/home outcomes of infants exposed to opioids in utero and controls. We are currently recruiting exposed and control infants at all four clinical sites and will provide important information on the impact of prenatal opioid exposure on the trajectory of brain development over the first 2 years of life.

### Impact of opioid exposure on the brain

Previous studies suggest that prenatal opioid exposure affects brain development. A small study of opioid-exposed infants (*n* = 29) and controls (*n* = 42) having MRI scans before 2 months of age found that exposed infants had significantly smaller relative volumes of deep gray matter, insular white matter, thalamic ventrolateral nuclei, subthalamic nuclei, brainstem, and cerebrospinal fluid and larger volumes of right cingulate gyrus white matter and left occipital lobe white matter as compared to unexposed infants.^10^ These volumetric changes appear to persist throughout childhood. Compared to individuals without prenatal exposure, quantitative MRIs in adolescents who were drug-exposed demonstrated smaller neuroanatomical volumes, cortical surface areas, and thinner cortices.^7^ In another small study,^55^ a significant proportion (40%) of opioid-exposed infants were found to have punctate white matter lesions or white matter signal abnormality on MRI before 2 months of age, suggesting that opioid exposure is related to aberrant white matter development or even injury. Opioid exposure has also been associated with altered structural (as measured by DTI)^11^ and functional (as measured by resting-state functional connectivity MRI) connectivity.^12,13^ However, all previous studies in this area have been small and have not included well-matched control groups, underscoring the importance of the OBOE study.

### Impact of opioid exposure on development, behavior, and vision

Prenatal opioid exposure may affect later development, behavior, and vision. Several recent reviews and meta-analyses have concluded that infants exposed to opioids in utero are at higher risk of cognitive, language, and visual delays and behavior problems.^56–61^ However, most of these articles have concluded that these findings may be the result of bias in the populations studied and poor quality studies, with particular imbalances in the rate of tobacco exposure in opioid-exposed children versus controls.^56^ The OBOE study should overcome some of these limitations of prior research by including a control group prospectively recruited from the same birth hospitals as the exposed infants, with the inclusion of infants with prenatal nicotine exposure in the control group. The rigorous measures of neurodevelopment including the Bayley Scales of Infant Development, fourth edition conducted by certified examiners and prospective vision screening at age 2 years will also overcome limitations in rigor of prior research.

### Role of parenting and the home environment

Several studies have shown that after controlling for social–environmental and socioeconomic variables, the impact of in utero exposure to various substances on developmental and behavioral outcomes is no longer significant. When examining the outcomes of preschool children with antenatal cocaine exposure, the quality of the caregiving environment was the strongest independent predictor of all outcomes.^62^ Early research examining opioid exposure specifically demonstrated that controlling for social–environmental risk factors such as lower maternal education, low SES, and poor maternal–infant interactions eliminated the significant impact of early exposure on cognitive development.^63^ Furthermore, in the large-scale Maternal Lifestyles Study, the effects of opioid exposure on motor development were no longer significant after controlling for covariates such as SES and quality of the home environment in children through 3 years of age.^64^ The OBOE study was designed to collect extensive information on caregiver IQ (WASI-II), the home environment (HOME Inventory), parenting (MPAQ, HOME), maternal stress/depression/anxiety (PSI-4, PROMIS measures), and social determinants of health (demographic questionnaires).

## Conclusions

With the current opioid crisis in the U.S. and the increasing number of infants with NOWS, more information is needed on the longer-term outcomes of antenatal opioid exposure. The OBOE study will address limitations of prior research to further scientific knowledge of the effects of antenatal opioid exposure on infant brain development and neurodevelopmental outcomes through the use of serial neuroimaging and comprehensive assessments. The study findings can inform legislators and policy makers in decision-making on health policies, interventions, and resources needed to support the development of children born with antenatal opioid exposure.

## Data Availability

Upon completion of the study, data will be available through the NICHD Data and Specimen Hub (DASH).
