# Correction To: Outcomes of Babies with Opioid Exposure (OBOE): protocol of a prospective longitudinal cohort study

**DOI:** 10.1038/s41390-023-02662-7

**Published:** 2023-05-24

**Authors:** Carla M. Bann, Jamie E. Newman, Brenda Poindexter, Katherine Okoniewski, Sara DeMauro, Scott A. Lorch, Deanne Wilson-Costello, Namasivayam Ambalavanan, Myriam Peralta-Carcelen, Catherine Limperopoulos, Kushal Kapse, Jonathan M. Davis, Michele Walsh, Stephanie Merhar

**Affiliations:** 1 RTI International, Research Triangle Park, NC USA; 2 Emory University, Atlanta, GA USA; 3 Children’s Hospital of Philadelphia, Philadelphia, PA USA; 4 Case Western Reserve University, Cleveland, OH USA; 5 University of Alabama, Birmingham, AL USA; 6 Children’s National Medical Center, Washington, DC USA; 7 Tufts University, Boston, MA USA; 8 Eunice Kennedy Shriver National Institute of Child Health and Human Development, Bethesda, MD USA; 9 Cincinnati Children’s Hospital Medical Center and University of Cincinnati Department of Pediatrics, Cincinnati, OH USA

Correction to: *Pediatric Research* 10.1038/s41390-022-02279-2, published online 30 August 2022

The article “Outcomes of Babies with Opioid Exposure (OBOE): protocol of a prospective longitudinal cohort study”, written by Carla M. Bann, Jamie E. Newman, Brenda Poindexter, Katherine Okoniewski, Sara DeMauro, Scott A. Lorch, Deanne Wilson-Costello, Namasivayam Ambalavanan, Myriam Peralta-Carcelen, Catherine Limperopoulos, Kushal Kapse, Jonathan M. Davis, Michele Walsh and Stephanie Merhar, was originally published Online First without Open Access. After publication in volume 93 issue 6, page 1772–1779 the author decided to opt for Open Choice and to make the article an Open Access publication. Therefore, the copyright of the article has been changed to ©The Author(s) 2023 and the article is forthwith distributed under the terms of the Creative Commons Attribution 4.0 International License, which permits use, sharing, adaptation, distribution and reproduction in any medium or format, as long as you give appropriate credit to the original author(s) and the source, provide a link to the Creative Commons licence, and indicate if changes were made. The images or other third party material in this article are included in the article’s Creative Commons licence, unless indicated otherwise in a credit line to the material. If material is not included in the article’s Creative Commons licence and your intended use is not permitted by statutory regulation or exceeds the permitted use, you will need to obtain permission directly from the copyright holder. To view a copy of this licence, visit http://creativecommons.org/licenses/by/4.0/.

